# Novel Genetic Variants Explaining Severe Adverse Drug Events after Clinical Implementation of *DPYD* Genotype-Guided Therapy with Fluoropyrimidines: An Observational Study

**DOI:** 10.3390/pharmaceutics16070956

**Published:** 2024-07-19

**Authors:** Xando Díaz-Villamarín, María Martínez-Pérez, María Teresa Nieto-Sánchez, Gabriela Ruiz-Tueros, Emilio Fernández-Varón, Alicia Torres-García, Beatriz González Astorga, Isabel Blancas, Antonio J. Iáñez, José Cabeza-Barrera, Rocío Morón

**Affiliations:** 1 Instituto de Investigación Biosanitaria de Granada (Ibs.Granada), 18012 Granada, Spain; 2 Hospital Pharmacy, Hospital Universitario San Cecilio, 18016 Granada, Spain; 3 Department of Pharmacology, Center for Biomedical Research (CIBM), University of Granada, 18016 Granada, Spain; 4 Medical Oncology, Hospital Universitario San Cecilio, 18016 Granada, Spain; 5 Hospital Pharmacy, Hospital Regional Universitario de Málaga, 29010 Málaga, Spain

**Keywords:** fluoropyrimidines, *DPYD*, personalized medicine, pharmacogenetics, clinical implementation

## Abstract

Fluoropyrimidines (FPs) are commonly prescribed in many cancer streams. The EMA and FDA-approved drug labels for FPs recommend genotyping the DPYD*2A (rs3918290), *13 (rs55886062), *HapB3 (rs56038477), alleles, and *DPYD* rs67376798 before treatment starts. We implemented the *DPYD* genotyping in our daily clinical routine, but we still found patients showing severe adverse drug events (ADEs) to FPs. We studied among these patients the *DPYD* rs1801265, rs17376848, rs1801159, rs1801160, rs1801158, and rs2297595 as explanatory candidates of the interindividual differences for FP-related toxicities, examining the association with the response to FPs . We also studied the impact of *DPYD* testing for FP dose tailoring in our clinical practice and characterized the *DPYD* gene in our population. We found a total acceptance among physicians of therapeutic recommendations translated from the *DPYD* test, and this dose tailoring does not affect the treatment efficacy. We also found that the DPYD*4 (defined by rs1801158) allele is associated with a higher risk of ADEs (severity grade ≥ 3) in both the univariate (O.R. = 5.66; 95% C.I. = 1.35–23.67; *p* = 0.014) and multivariate analyses (O.R. = 5.73; 95% C.I. = 1.41–28.77; *p* = 0.019) among FP-treated patients based on the *DPYD* genotype. This makes it a candidate variant for implementation in clinical practice.

## 1. Introduction

Fluorouracil and its oral prodrug capecitabine, both fluoropyrimidines (FPs), are anticancer drugs used in the treatment of many solid tumors, such as breast and gastric, but especially in colorectal cancer. Despite the wide use of these drugs, among FP-treated patients with standard doses, up to 30% have severe (grade ≥ 3) treatment-related toxicity [1,2], which can lead to treatment-related death in up to 1% of patients [2,3,4]. The most common severe treatment-related toxicity includes diarrhea, oropharyngeal mucositis, hand–foot syndrome, and myelosuppression [2,5].

**Figure 1:**
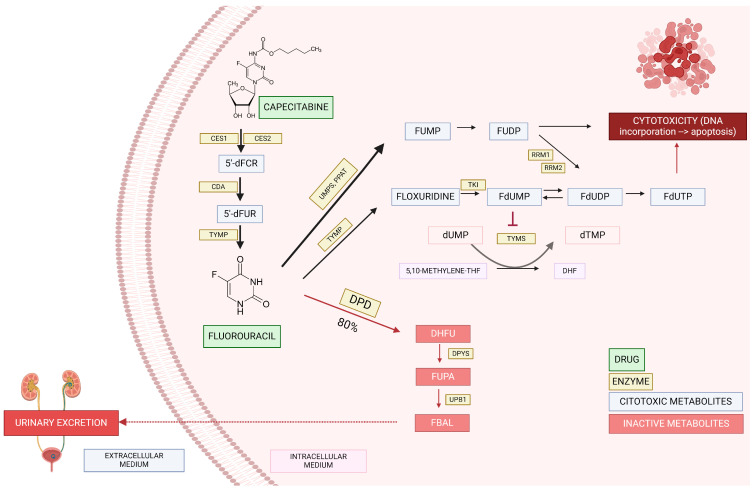
Capecitabine/fluorouracil pathway. Carboxylesterase 1 (CES1); carboxylesterase 2 (CES2); cytidine deaminase (CDA); thymidine phosphorylase (TYMP); 5′-deoxy-5-fluorocytidin (5′dFCR); 5′-deoxy-5-fluorouridine (5′dFUR); uridine-5-monophosphate synthase (UMPS); amidophosphoribosyltransferase (PPAT); dihydropyrimidine dehydrogenase (DPD); fluorouridine monophosphate (FUMP); fluorouridine diphosphate (FUDP); ribonucleoside diphosphate reductase (RRM1 y 2); thymidine kinase (TKI); fluorodeoxyuridine monophosphate (FdUMP); fluorodeoxyuridine diphosphate (FdUDP); fluorodeoxyuridine triphosphate (FdUTP); deoxyuridine monophosphate (dUMP); deoxythymidine monophosphate (dTMP); 5,10 methylene tetrahydrofolate (5–10 methylene THF); dihidrofolato (DHF); dihydrofluorouracil (DHFU); dihidropirimidinasa (DPYS); 5-fluoro-ureidopropionic acid (FUPA); β-ureidopropionase (UPB1); α-fluoro-β-alanina (FBAL).

Fluorouracil is administered intravenously, while capecitabine is an oral prodrug metabolized by carboxylesterase, cytidine deaminase, and thymidine phosphorylase resulting, respectively, in 5′-deoxy-5-fluorocytidin (5′dFCR), 5′-deoxy-5-fluorouridine (5′dFUR), and finally 5-fluorouracil (5-FU) [6] (Figure 1). After this, around 80% of fluorouracil is inactivated, and the remaining 20% is converted into cytotoxic metabolites or excreted in the urine [5]. FPs are catabolized by different enzymes, leading to their inactivation, and dihydropyrimidine dehydrogenase (DPD) is the main enzyme responsible for this [7].

Patients with reduced DPD activity catabolize less fluorouracil, leading to more than 20% of cytotoxic/therapeutic metabolites, thus increasing the risk of supratherapeutic drug availability toxicity. In European and North American patients, reduced and complete lack of DPD activity are present in 3–5% and 0.01–0.1%, respectively [2].

### 1.1. The Dihydropyrimidine Dehydrogenase Gene (DPYD)

Many genetic variants in the gene encoding the DPD enzyme (*DPYD* gene) partially explained the interindividual differences in the toxicity of FPs. The most relevant genetic variants in this regard are the *DPYD* rs67376798 (c.2846A>T, D949V), the rs3918290 (c.1905+1G>A, IVS14+1G>A) defining the DPYD*2A allele, the rs55886062 (c.1679T>G, I560S) defining the DPYD*13, and the rs75017182 (c.1129-5923C>G) and rs56038477 (c.1236G>A, E412E) characterizing the DPYD*haplotype (Hap) B3. All of them have been associated with a reduction in DPD activity, resulting in higher concentrations of fluorouracil cytotoxic metabolites and an increased risk of severe or fatal toxicities.

The DPYD*2A (rs3918290) was the first allele known to have a functional impact on DPD activity [8,9]. It was stated that homozygous carriers of this variant have a complete loss of function of DPD while heterozygous carriers showed a significant reduction in mean DPD activity [9,10]. Several studies reported a significant association of this allele with the toxicity of FPs, even though the FP dose tailoring based on DPYD*2A genotype was demonstrated to be an effective strategy for the prevention of severe or fatal toxicity events [11]. In their study, Deene et al. showed in a cohort of n = 1631 patients that a 50% dose reduction in DPYD*2A (rs3918290) carriers may decrease the FP-related toxicity from 73% to 28% (*p* < 0.001). Thereafter, when studying DPYD*2A (defined by rs3918290) combined with DPYD*13 (defined by rs55886062), DPYD*Hap B3 (defined by rs56038477), and rs67376798, a 25.6-fold increased risk of death in FP-treated patients was found [12], and a systematic review and meta-analysis including n = 7365 patients concluded that *DPYD* genotyping for these four *DPYD* variants can identify a significant ratio of patients who are DPD deficient [13]. Finally, Henricks L.M. et al. [2] found the prospective *DPYD* genotyping feasible in routine clinical practice, and dose reductions based on *DPYD* genotype improved patient safety in FP-treated patients. In this prospective multicenter study, they tailored the FP treatment dose based on DPYD*2A (defined by rs3918290), DPYD*13 (defined by rs55886062), DPYD*HapB3 (defined by rs56038477), and rs67376798 in n = 1103 patients. DPYD*2A and *13 carriers received a 50% dose reduction, while rs67376798 and DPYD*HapB3 carriers received a dose reduction of 25%. Results indicated that FP-related severe toxicity (grade ≥ 3) was more prevalent among *DPYD* variant carriers compared to wild-type patients (*p* = 0.0013), and a significantly lower relative risk for severe FP-related toxicity for *DPYD* genotype-guided dosing was observed compared with historical cohorts for each genetic variant carrier.

On the other hand, DPYD rs3918290, rs55886062, and rs56038477, characterizing the DPYD*2A, *13, and *HapB3 alleles, respectively, and rs67376798, are not the only DPYD variants associated with the toxicity of FPs. There are many others that showed the highest level of evidence according to the PharmGKB [14] with this association (Table 1).

### 1.2. Pharmacogenetics of Fluoropyrimidines

All the evidence regarding the association of *DPYD* variants with FP-related toxicity, including the reduction in toxicity with dose tailoring based on the *DPYD* genotype and the clinical impact of this practice, has led to updates for the drug labels approved by the Food and Drug Administration (FDA) and the European Medicines Agency (EMA), incorporating this information, as well as the development of pharmacogenetic (PGx) dosing guidelines for these drugs.

The European Public Assessment Report (EPAR) [16] for capecitabine by the EMA states that capecitabine is contraindicated in patients with no DPD activity and recommends a reduction in starting dose in reduced DPD activity patients to avoid serious toxicity. The EPAR also reports that the reduced DPD activity may be stated considering the *DPYD* genotype and highlights DPYD*2A (defined by rs3918290), DPYD*13 (defined by rs55886062), DPYD*HapB3 (defined by rs56038477), and rs67376798 (also known as c.2846A>T) as being mainly responsible for DPD reduced/complete absence activity, while considering that there may be other *DPYD* variants associated with an increased risk of life-threatening toxicity. The FDA table of PGx [17] includes information about the capecitabine and fluorouracil/*DPYD* drug–gene interactions, reporting that genotype-translated DPD intermediate or poor metabolizer (IM or PM) phenotypes result in higher severe or life-threatening risk toxicities. It considers that there is no dosage safe in PMs and recommends withholding or discontinuing treatment in the presence of early-onset or unusually severe toxicity.

Carrying these *DPYD* variants alone or in combination results in different translated phenotypes for DPD activity, thus different dosing recommendations. As commented above, there are available PGx dosing guidelines including this information. Both the Clinical Pharmacogenetics Implementation Consortium (CPIC) [18] and Dutch Pharmacogenomics Working Group (DPWG) guidelines [19] categorize patients’ *DPYD* gene activity score (GAS) depending on *DPYD* genotype, as shown in Table 2. Furthermore, CPIC guidelines refer to the DPD metabolizing status and categorize patients as normal metabolizers (NM) if they are not carrying any of these variants (GAS = 2), as IM if *DPYD* GAS = 1 or 1.5, and as PM when the GAS is lower than 1.

Also, both the DPWG and CPIC guidelines (Table 3), recommend an alternative drug, if possible, in patients with a *DPYD* GAS lower than 1 (DPD PMs, in CPIC guidelines), and to start with 50% of the standard dose in patients with a GAS of 1 or 1.5 (DPD IMs). Also, the CPIC guideline reports that IM patients carrying the c.[2846A>T]/[2846A>T] genotype (GAS = 1) may require a dose reduction higher than 50%. The main difference between the CPIC and DPWG guidelines is the *DPYD* alleles considered for analysis. While the DPWG considers only the DPYD*2A (defined by rs3918290), DPYD*13 (defined by rs55886062), DPYD*HapB3 (defined by rs56038477), and rs67376798 (also known as c.2846A>T), the CPIC guideline for FPs and *DPYD* provides a list with n = 83 variants, resulting in diplotypes translated into IM or PM (GAS < 2) phenotypes for reduced DPD activity and FP dose tailoring.

### 1.3. Hypothesis and Objectives

The EMA, AEMPS, SEFF, SEOM, and the drug labels for capecitabine and 5-FU recommend *DPYD* genotyping to state the DPD metabolizing status before treatment starts with these drugs. Furthermore, there are available dosing guidelines based on PGx information, such as those from the DPWG [19] and CPIC [18].

We have implemented *DPYD* genotyping in our daily clinical practice before treatment starts with FPs. Despite this, many patients still show severe adverse drug events.

With this study, we aim to state the impact in our clinical routine of capecitabine and 5-FU dose tailoring based on DPYD*2A (defined by rs3918290), DPYD*13 (defined by rs55886062), DPYD*HapB3 (defined by rs56038477), and rs67376798 (also known as c.2846A>T), studying its association with the treatment efficacy and toxicity.

Also, we aim to explain the toxicity events in the cohort of patients receiving the FP treatment tailored by *DPYD* genotype. In this regard, we studied the association with the FP toxicity and efficacy of other relevant *DPYD* variants, previously associated with FP response, but not recommended by the scientific societies and sanitary authorities.

Finally, we aimed to characterize the *DPYD* gene in our population, studying its genotypes and phenotypes distribution, the minor allele frequency (MAF), Hardy–Weinberg (H-W) equilibrium of included variants, and the possible linkage disequilibrium (LD).

In Spain, the Spanish Agency for Medicine and Health Products (*Agencia Española del Medicamento y Productos Sanitarios*, AEMPS), Spanish Society of Pharmacogenetics and Pharmacogenomics (*Sociedad Española de Farmacogenética y Farmacogenómica,* SEFF), and Spanish Society of Medical Oncology (*Sociedad Española de Oncología Médica*, SEOM), recommend genotyping the DPYD*2A (defined by rs3918290), DPYD*13 (defined by rs55886062), DPYD*HapB3 (defined by rs56038477), and rs67376798 (also known as c.2846A>T) prior to FP treatment, avoiding its use in DPD PM patients (GAS < 1), and using a 50% dose reduction in DPD IM patients (1 < GAS < 2).

Since 2021, we have implemented the PGx test of DPYD*2A (defined by rs3918290), DPYD*13 (defined by rs55886062), DPYD*HapB3 (defined by rs56038477), and rs67376798 (also known as c.2846A>T) in our hospital before capecitabine and fluorouracil treatment initiation, as well as dose tailoring based on guidelines from the DPWG. Despite this implementation in clinical practice and its acceptance by sanitary authorities and physicians in daily routine, we still have patients experiencing severe or life-threatening FP-related toxicities.

## 2. Materials and Methods

### 2.1. Study Design

The observational retrospective study including patients treated with capecitabine or fluorouracil and tested for DPYD*2A (defined by rs3918290), DPYD*13 (defined by rs55886062), DPYD*HapB3 (defined by rs56038477), and rs67376798 (also known as c.2846A>T) before treatment began in our hospital between 1 March 2021 and 31 December 2021.

The inclusion criteria were patients diagnosed with cancer and prescribed capecitabine or fluorouracil, those tested for DPYD*2A (defined by rs3918290), DPYD*13 (defined by rs55886062), DPYD*HapB3 (defined by rs56038477), and rs67376798, with a capecitabine or fluorouracil dose tailored based on DPWG guidelines, with an available 6-month follow-up period based on medical records, and non-previously treated with FPs. Patients who did not sign the informed consent form or who asked for to withdraw from the study were excluded.

The Research Ethics Committee of Granada approved the study (Code: 1605-N_22; date of approval: 14 September 2022) and the principles of the Declaration of Helsinki were followed.

The main endpoints were the toxicity and efficacy of capecitabine or 5-FU. These data was obtained from electronic medical records and confirmed by physicians in case of discrepancies.

The toxicity endpoint was adverse drug events (ADEs) to capecitabine or 5-FU. The causality and severity of ADEs were stated using the Liverpool Causality Assessment Tool (LCAT) [20], and Common Terminology Criteria for Adverse Events (CTCAE) [21], respectively. Only ADEs categorized as probable or definite and showing a severity grade 3 or higher were considered for the study.

The efficacy endpoint was achieved if patients received a positive clinical assessment from the oncologist regarding the progression of the illness, as recorded in the medical records, and the non-discontinuation of FP treatment during follow-up if it was not because of an ADE. For the positive clinical assessment of metastatic patients by the oncologists, the Response Evaluation Criteria in Solid Tumors (RECIST 1.1) measure was used within 6 months of initiating therapy [22]. Radiological and imaging techniques, such as computed tomography (CT), were employed to monitor the evolution of both target and non-target lesions in treated patients. These assessments were conducted every 3 to 4 months (1–2 times during follow-up), unless patients exhibited clinical signs of progression earlier (e.g., pain, dyspnea, sweating, elevated tumor markers in blood tests).

### 2.2. Procedures for the Inclusion of DPYD Variants in the Study

To include candidate *DPYD* variants as explanatory factors of remaining toxicity events in our study population, who were receiving dose-tailored FP treatment based on DPYD*2A (defined by rs3918290), DPYD*13 (defined by rs55886062), DPYD*HapB3 (defined by rs56038477), and rs67376798, we searched in PharmGKB [14] for genetic variants of *DPYD* reported as clinical annotations with the highest level of evidence (1A) concerning their association with any phenotype for capecitabine, 5-FU, or tegafur (Table 1). We included those genetic variants related to the toxicity of these drugs with MAF values higher than 1% in the Iberian Peninsula population according to the 1000 Genomes project [15].

### 2.3. Management of Patients

As part of the clinical practice at our hospital, whenever a doctor considers prescribing capecitabine or 5-FU in patients diagnosed with whatever cancer, they may request a *DPYD* PGx test from the hospital pharmacy.

Once the request for the test is received, a nurse takes a saliva sample with sterile cotton swabs, the DNA is extracted, and DPYD*2A (defined by rs3918290), DPYD*13 (defined by rs55886062), DPYD*HapB3 (defined by rs56038477), and rs67376798 are tested less than 48 h after the saliva sample collection. The pharmacists upload a PGx report in the electronic medical records, including the therapeutic recommendation translated from the PGx result, within 72 h of sample collection.

The genotype–phenotype–therapeutic recommendation translation process (dose tailoring) is performed following the instructions in the DPWG guidelines and is shown in Table 2 and Table 3. This means that patients not carrying any of the tested variants are assigned a *DPYD* GAS = 2 and treated with the standard dose. Those carrying a single mutated allele are assigned GAS = 1–1.5 and start the treatment with a 50% reduction in the standard dose. Patients with two mutated alleles are assigned GAS = 0, and the treatment is switched.

The remaining DNA and saliva samples are stored as a private biosamples collection registered with the Carlos III Health Institute (C.0007322). Once the follow-up of patients was finished, we retrospectively tested, using this remaining DNA, the *DPYD* variants meeting the criteria that are explained in Section 2.2.

Those patients not treated with capecitabine or fluorouracil after the PGx test were excluded from the analysis. The included patients were followed up for six months.

### 2.4. Data Management, Statistical Analysis, and Genotyping

First, a descriptive analysis of the population included in the study was performed. The impact and usefulness of the *DPYD* PGx test for capecitabine and 5-FU dose tailoring in our clinical practice was assessed by studying the association with the toxicity and efficacy endpoints of the dose tailoring based on DPYD*2A (defined by rs3918290), DPYD*13 (defined by rs55886062), DPYD*HapB3 (defined by rs56038477), and rs67376798.

After that, to explain ADEs that occurred during follow-up in patients receiving an FP-*DPYD*-dose tailored treatment, we performed a genotype association study with the toxicity and efficacy endpoint.

Moreover, we carried out a multivariate analysis to discard possible confounding factors on the association of genetic variants with the toxicity and efficacy endpoints.

Finally, we characterized the *DPYD* variants in our population, including both those used in our daily clinical practice and those studied for their association with the toxicity endpoint after PGx FP dose tailoring. In this regard, the distribution (number, n, and percentage, %) of genotypes, phenotypes, and MAFs were calculated, an LD analysis was carried out, and the H-W equilibrium was tested.

The descriptive analysis, MAFs, genotype/phenotype distribution, and multivariate analysis were conducted using R commander. For the association studies of genetic variants with the endpoints, LD analysis, and H–W equilibrium analyses, we used the SNPstats online tool [23].

For the multivariate analysis, we considered for inclusion all the study variables recorded, including clinical parameters, genetic variants, and concomitant treatments. We built different multivariate models using the backward, forward, and stepwise methods for the association with the efficacy and toxicity endpoints. These models were upgraded and compared using the Akaike information criterion (AIC). The final model showing the lower AIC was chosen.

The chi-square test or Fisher exact test were used, and odds ratios (OR) and *p*-values were calculated, considering *p* < 0.05 to be statistically significant.

### 2.5. DNA Extraction and Genotyping

For genotyping, DNA was isolated from saliva samples using standard procedures. DNA extraction was carried out following the method by Freeman et al. [24], a non-organic (proteinase K and salting out) protocol including modifications from the method described by Gomez-Martín A. et al. [25]. The included genetic variants were genotyped using Taqman assay technology (Thermo Fisher Scientific, Waltham, MA, USA) and analyzed with QuantStudio 12K Flex de Applied Biosystems (Foster City, CA, USA).

## 3. Results

**Figure 2:**
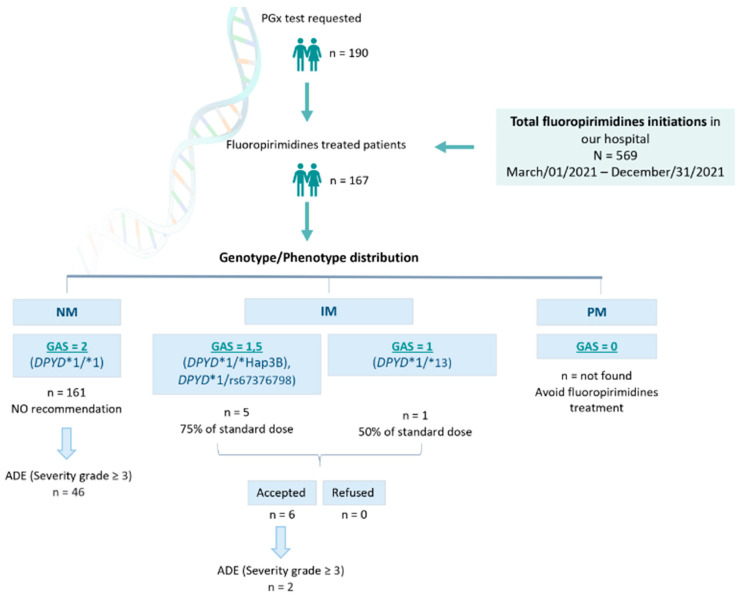
Recruitment and management of patients. PGx: pharmacogenetic; NM: normal metabolizer; IM: intermediate metabolizer; PM: poor metabolizer; GAS: Gene Activity Score; ADE: adverse drug event.

Between Mar/01/2021 and Dec/31/2021, N = 569 patients were prescribed capecitabine or 5-FU at the *Hospital Universitario Clínico San Cecilio* (Granada, Spain). In total, n = 190 *DPYD* PGx tests were requested, and n = 167 patients were finally treated with FPs (Figure 2). This means n = 402 patients were prescribed capecitabine or 5-FU in our hospital without being *DPYD* tested, and n = 23 were *DPYD* tested but not treated with FPs.

Among the n = 167 *DPYD*-tested patients finally treated with capecitabine or 5-FU, we found n = 161 DPYD*1/* 1 (wild-type) patients, translated into a GAS = 2 phenotype receiving normal doses of FPs. We also found n = 1 DPYD*1/*13, n = 3 *1/*HapB3, and n = 2 *1/c.2846A>T genotypes, translated into n = 5 GAS = 1.5, and n = 1 GAS = 1, who were recommended to be treated with 50% of the standard dose. All these patients were dose-adjusted based on our recommendation (Figure 2). Among those n = 23 non-treated patients, we found n = 21 DPYD*1/*1 (wild-type) and n = 2 *1/*HapB3.

All the n = 167 FP-treated patients after dose tailoring based on *DPYD* genotyping were prescribed treatment because of digestive tumors, including colon (n = 81), stomach (n = 13), pancreas (n = 10), duodenum (n = 1), esophageal (n = 1), and rectal cancer (n = 45), although breast cancer (n = 12) and other kind of tumors (n = 4) affected some patients. All the patients received capecitabine or 5-FU as a first-line treatment, except the n = 12 breast cancer patients who were prescribed it after cytotoxic chemotherapy failure and a locally advanced tumor or metastasis. The mean age was 64.26  ±  10.89 years old, and 37.13% were women (Table 4).

### 3.1. Association Study of DPYD Variants with the Response

#### 3.1.1. Association with Response of Dose Tailoring Based on DPYD

We found no association between the *DPYD* variants used for FP dose tailoring and the toxicity endpoint. We also found no association with the efficacy endpoint (Table 5). This means that carrying a genotype translated into a GAS lower than 2 (DPD IM or PM) and receiving adjusted doses is not related to variable response to FPs.

#### 3.1.2. Association with the Response of New DPYD Variants

Among treated patients based on DPYD*2A (defined by rs3918290), DPYD*13 (defined by rs55886062), DPYD*HapB3 (defined by rs56038477), and rs67376798, we still found n = 47 patients meeting the toxicity endpoint.

As commented above, we retrospectively studied n = 6 *DPYD* variants that had been related to the toxicity of FPs and with an MAF higher than 1% in the Iberian Peninsula population according to the 1000 Genomes project.

We found that carrying the *DPYD* rs1801158 is associated with a higher risk of ADEs with a severity grade ≥ 3 (OR = 5.66; 95% C.I. = 1.35–23.67; *p* = 0.014) (Table 6). No other *DPYD* variant was associated with the toxicity endpoint.

We found no association of any of the included SNPs with the efficacy endpoint.

#### 3.1.3. Multivariate Analysis

The following variables were included as possible explanatory parameters of the efficacy and toxicity endpoints in the multivariate analysis: clinical variables, including age, sex, and body surface area; concomitant drugs in the therapeutic scheme, including monoclonal antibodies, oxaliplatin, irinotecan, and radiotherapy; and all the *DPYD* genetic variants included in the study, i.e., those used and not used for FP dose tailoring.

For the multivariate analysis to explain the toxicity endpoint, the model using the stepwise (backward/forward) method showed the lower AIC, and this model was finally included. After adjustment, the model showed that the *DPYD* rs1801158 is associated with ADEs (severity grade ≥ 3) for FPs (OR = 5.73; 95% CI = 1.41–28.77; *p* = 0.019) in *DPYD* dose-tailored patients based on DPYD*2A (defined by rs3918290), DPYD*13 (defined by rs55886062), DPYD*HapB3 (defined by rs56038477), and rs67376798 (Table 7).

In the multivariate analysis to explain the efficacy endpoint we found that the concomitant treatment with irinotecan is associated with lower rates of efficacy (*p* ≤ 0.001).

### 3.2. DPYD Characterization

In total, n = 190 patients were requested to be tested and dose-tailored based on DPYD*2A (defined by rs3918290), DPYD*13 (defined by rs55886062), DPYD*HapB3 (defined by rs56038477), and rs67376798. Among them, n = 189 were tested for *DPYD* variants considered candidates to explain differences in the response to FPs. This means that n = 1 patient could not be tested for these variants because we did not have enough stored DNA.

Among all the tested variants in our population, we found no differences with the MAF for the Iberian Peninsula population reported by the 1000 Genomes Project [15], and all the SNPs were in the Hardy–Weinberg equilibrium (Table 8).

We found many significant differences in the LD analysis. The *DPYD* rs1801265 was linked to the rs56038477 (r = 0.214; *p* < 0.001) and the rs2297595 (r = 0.604; *p* < 0.001) in our population (Table 9). In more detail, we found that all the patients carrying rs56038477 also carried rs1801265, and n = 31 (83.8%) of patients carrying rs2297595 also carried rs1801265. We found other *p*-values lower than 0.05 in the LD analyses but showing r values close to r = 1.

## 4. Discussion

FPs, including 5-FU and the oral prodrug capecitabine, are commonly prescribed antimetabolite chemotherapies utilized across many cancer streams.

Among FP-treated patients with standard doses, up to 30% show severe (grade ≥ 3) treatment-related toxicity. Many genetic variants in the *DPYD* gene encoding the DPD enzyme partially explained these toxicities. The DPYD*2A (defined by rs3918290), *13 (defined by rs55886062), *HapB3 (defined by rs56038477), alleles, and *DPYD* rs67376798 had shown the highest level of evidence about their association with FPs response. The EMA- and FDA-approved drug labels for FPs, and the SEFF and SEOM in Spain, recommend genotyping these variants before treatment starts.

Depending on *DPYD* genotype, patients may be categorized as DPD NM, IM, or PM and receive a PGx dose-tailored treatment (50% of standard doses in DPD IM patients, and alternative therapies in DPD PMs).

Moreover, different studies concluded that dose adjustments based on *DPYD* genotype do not influence the treatment efficacy. Deenen M.J. et al. did not find a relationship between *DPYD* variants and progression-free survival or overall survival despite a 50% dose reduction in DPYD*2A carriers [26], and Lam S.W. et al. [27] observed no differences in response in seven more clinical studies examining *DPYD* polymorphisms with a dose reduction, time to progression, progression-free survival, and/or overall survival, concluding that there is no evidence that a priori dose adjustments for *DPYD* carriers decrease FP efficacy, and low-activity variant carriers treated with standard of care appear to have similar efficacy once an acceptable dose is found.

We implemented *DPYD* genotyping in our daily clinical routine, but we still find patients showing severe toxicities to FPs. Thus, we hypothesized that there might be other variants influencing the FP-related toxicities.

We identified six *DPYD* variants (rs1801265, rs17376848, rs1801159, rs1801160, rs1801158, and rs2297595) as explanatory candidates of the interindividual differences for the FP-related toxicities, since these had been related to the toxicity of FPs with the highest level of evidence, and they have an MAF higher than 1% in the Iberian Peninsula population.

In this study, we assessed the association with response to FPs of these novel candidate variants to explain suboptimal patient response for the first time in a cohort that received FP treatment based on DPYD*2A (defined by rs3918290), *13 (defined by rs55886062), *HapB3 (defined by rs56038477), and rs67376798.

This way, we could determine whether these new *DPYD* variants explain the remaining toxicities despite a PGx dose-tailored treatment and whether they would be potentially useful in daily clinical practice.

This study may also be used as a guide for the clinical implementation of the FP-*DPYD* drug–gene interaction.

### 4.1. Association of Genetic Variants with Response to Fluoropyrimidines

In the study of the association of FP dose tailoring according to the *DPYD* genotype with the toxicity and efficacy endpoints, we found no significant differences (Table 5). These results make sense, since significant differences would have meant an underestimation of dose modifications resulting from the presence of the DPYD*2A (defined by rs3918290), DPYD*13 (defined by rs55886062), DPYD*HapB3 (defined by rs56038477), and rs67376798 variants.

In the association study with the toxicity and efficacy endpoints of the new variants, which are not currently being used to guide treatment with FPs in our population, we have observed the following.

The DPYD*4 (defined by rs1801158) allele is associated with a higher risk of severe ADEs (severity grade ≥ 3) in both the univariate (OR = 5.66; 95% CI = 1.35–23.67; *p* = 0.014) and multivariate analyses (OR= 5.73; 95% CI= 1.41–28.77; *p* = 0.019) after adjusting the model.

This variant had been previously associated with FP response. The DPYD*4 CT genotype was associated with decreased catalytic activity of DPD [28], and an increased risk of drug toxicity when treated with capecitabine or fluorouracil in colorectal cancer patients [29], as compared to CC genotype. On the other hand, many other studies showed contradictory results [13] in this regard. An interesting study by André B. P. van Kuilenburg et al. [30] found that DPYD*4 allele T is associated with decreased activity of DPD when expressed in mammalian cells (HEK293 Flp-In) as compared to allele C, while highlighting the conflicting data about this association, as they found no association when DPD activity was assessed within a healthy cohort of n = 100 individuals.

These findings, and the results described below, reveal the need for further studies, especially considering the expression of genes, and not just categorizing patients as carriers/non-carriers of single or combined *DPYD* variants.

We found an association of chemotherapy schemes including irinotecan with a lower efficacy in the multivariate analysis (Table 7). We observed that the n = 10 patients treated with FOLFIRINOX (including irinotecan) are the same n = 10 patients with pancreatic cancer, with the worse baseline condition and prognosis among recruited patients.

Also, regarding our results, no other variants were significantly associated with either toxicity or efficacy endpoints. The *DPYD* rs17376848 showed a confounding association with the response. According to the significance criteria based on *p*-values, carriers of this variant (Genotype AG or GG vs. AA) were associated with a lower risk of the toxicity endpoint (*p* = 0.043) and a certain trend toward the efficacy of FPs (*p* = 0.079). However, upon closer examination of these results, we find that only n = 6 patients carry the *DPYD* rs17376848 variant, and none of them experienced an ADE (severity grade ≥ 3) during follow-up, preventing us from confirming this association. Furthermore, this statistical significance is lost in the multivariate analysis (*p* = 0.992; Table 7) when considering the influence of concomitant treatments, other clinical variables, and interactions with other *DPYD* variants.

As happens with DPYD*4 (defined by rs1801158), those other five variants suggested as candidate variants to explain the remaining toxicity events in our population showed inconclusive results in previous studies [13,31]. In any case, further studies with larger cohorts and treatment guidance based on these variants are necessary to confirm their utility or lack of influence in clinical practice.

Anyway, despite of the baseline characteristics of recruited patients, stage of the tumor, different fluoropyrimidines schemes, interactions between clinical and PGx variables, and receiving a PGx dose tailored treatment, the DPYD*4 (defined by rs1801158) showed an association with a higher risk of severe ADEs with FPs in our population. This makes it a candidate variant for potential implementation in clinical practice.

### 4.2. Insights on Clinical Practice

We have observed that in the clinical practice of our hospital, there is an important association between the profile of the health professional and the degree of acceptance and requests for PGx tests. As also happened to us in previous studies [32], there is a bias between the hospital departments prescribing the drugs and those requesting the PGx tests. In this case, we have observed that 96.3% (n = 183) of the n = 190 requests for the *DPYD* test were made by the medical oncology department, and n = 7 (3.7%) were made by other units of our hospital. Furthermore, we observed that, of the n = 170 patients who were prescribed capecitabine or 5-FU in the medical oncology unit during the time of recruitment, n = 167 (98.2%) received the treatment as guided by the PGx test. On the other hand, n = 402 patients in total in our hospital received treatment with FPs without having been genotyped for the variants recommended on the drug label by the EMA, FDA, SEFF, and SEOM. Among these, only n = 3 were treated by doctors assigned to the medical oncology service, and n = 399 were treated by other units.

As we can see, despite the level of evidence regarding the *DPYD*–FP interaction and the recommendations on the drug label by health authorities and scientific societies, the degree of implementation of these tests is strongly linked to the profile of the healthcare professional and the internal procedures or protocols of their hospital department.

On the other hand, it is true that until a few months ago, PGx tests, including the *DPYD*–FP interaction described in this study, had not been included in the portfolio of common services of the national health system in Spain, which is the set of health procedures that must be available to any citizen. The recent update of the service portfolio institutionally supports the performance of PGx tests and invites health professionals to perform them.

Another aspect that we observed in our results is that the level of acceptance of the therapeutic recommendations that emerge from the PGx results is total, and that dose reductions do not translate into a decrease in the treatment’s efficacy. Furthermore, based on our results and, as mentioned above, *DPYD* rs1801158 could be implemented in clinical practice. This variant has a relatively high frequency (MAF = 0.032; 6% carriers) and was the only genetic or clinical variant that was associated with an increased risk of toxicity secondary to FPs in the univariate and multivariate analyses.

The *DPYD*–FP interaction, regardless of many contradictory results, has been demonstrated to be useful in clinical practice in different studies. Furthermore, recent studies have demonstrated the cost-effectiveness of PGx tests [33,34]. The potential benefit, actual related costs, and support by sanitary authorities should be enough reasons for the large-scale implementation of PGx tests, especially the *DPYD*–FP interaction.

### 4.3. Limitations

This is not a comparative clinical trial; rather, it is an observational cohort study. We did not consider all the variants in the *DPYD* gene. However, for the recruited cohort, it was not useful to study further variants. In fact, we examined all variants with a MAG higher than 1% in our population. Rare variants do not make sense in a cohort of n = 167 patients.

Nevertheless, it is necessary to investigate rare variants in larger cohorts to study the different interactions between them in detail. In particular, conducting an association study between *DPYD* haplotypes and their response to FPs would be especially interesting. Also, studying the epigenetics of the *DPYD* gene as a key gene in the PGx of FPs would be also interesting. This might elucidate discrepancies between association studies of *DPYD* variants with their FP response.

We did not study all the clinical variables that influence the response to FPs, especially the baseline condition of recruited patients, as commented above. Furthermore, this study is based on real-world data obtained from daily clinical practice, and we are limited by the reliable information collected in the patients’ medical records.

In the same regard, we recruited a wide range of different patients, including metastatic/adjuvant-treated patients, different stages of tumors, chemotherapy schemes, etc. Anyway, the aim was to perform the study considering real-world data based on our daily clinical practice, and we found significant differences concerning the influence of the DPYD*4 alle on FP toxicities.

Also, considering the inclusion criteria of candidate *DPYD* variants as explanatory factors of FP-related toxicities, we should study the *DPYD* rs75017182 variant (Table 1). It was not genotyped because it has a lower MAF thanthe *DPYD* rs56038477,and both variants charactere the DPYD*HapB3. 

## 5. Conclusions

Based on our results, we concluded that FP dose lowering based on the *DPYD* genotype does not affect the treatment efficacy. DPYD*4 (defined by the rs1801158) is associated with FP toxicity in patients receiving a PGx dose-tailored treatment based on DPYD*2A (defined by rs3918290), DPYD*13 (defined by rs55886062), DPYD*HapB3 (defined by rs56038477), and rs67376798. Based on this, DPYD*4 (defined by the rs1801158) is an explanatory factor of remaining ADEs among FP-treated and PGx dose-tailored patients, and its genotyping should be implemented in daily clinical practice.

## Figures and Tables

**Table 1:** *DPYD* variants associated with any fluoropyrimidine phenotype with a 1A level of evidence (obtained from PharmGKB [14]).

*Allele	*DPYD* Variant	Major Nucleotide Variation	MAF			Molecules	Toxicity ^
			Ibs	Europe	Global		
-	rs115232898	c.557A>G	100/0	100/0	99/1	Fluorouracil	x
-	rs148994843	c.1543G>A	No data	100/0	100/0	Fluorouracil	
-	rs17376848	c.1896T>C	98/2	96/4	94/5	Capecitabine/fluorouracil	x
*4	rs1801158	c.1601G>A	94/6	97/3	99/1	Capecitabine/fluorouracil	x
*5	rs1801159	c.1627A>G	79/21	81/19	82/18	Capecitabine/fluorouracil	x
*6	rs1801160	c.2194G>A	95/5	95/5	96/4	Capecitabine/fluorouracil	x
*9A	rs1801265	c.85T>C	79/21	79/21	74/26	Capecitabine/fluorouracil	x
*8	rs1801266	c.703C>T	No data	100/0	100/0	Fluorouracil	
*10	rs1801268	c.2983G>T	No data	100/0	100/0	Fluorouracil	
-	rs2297595	c.496A>G	88/12	88/12	94/6	Capecitabine/fluorouracil	x
*2A	rs3918290	c.1905+1G>A	100/0	99/1	99/1	Capecitabine/fluorouracil/tegafur	x
*13	rs55886062	c.1679T>G	100/0	100/0	100/0	Capecitabine/fluorouracil/tegafur	x
-	rs56005131	c.2303C>A	100/0	100/0	100/0	Fluorouracil	
*HapB3	rs56038477	c.1236G>A	98/2	98/2	99/1	Capecitabine/fluorouracil	x
-	rs59086055	c.1774C>T	100/0	100/0	100/0	Fluorouracil	x
-	rs67376798	c.2846A>T	100/0	99/1	99/1	Capecitabine/fluorouracil/tegafur	x
*3	rs72549303	c.1898del	No data	100/0	100/0	Fluorouracil	
*11	rs72549306	c.1003G>T	No data	100/0	100/0	Fluorouracil	
*7	rs72549309	c.299_302del	No data	100/0	100/0	Fluorouracil	
*HapB3	rs75017182	c.1129-5923C>G	98/2	98/2	99/1	Capecitabine/fluorouracil	x
*12	rs78060119	c.1156G>T	No data	100/0	100/0	Fluorouracil	x

MAF: Minor allele frequency; Ibs: Iberian Peninsula; * MAFs obtained from the 1000 Genomes Project [15]. ^ An “x” means that the variant in that row has been associated with the toxicity of drugs reported in the column “molecules”.

**Table 2:** Phenotype translation from the considered *DPYD* allele combinations for fluoropyrimidine dose tailoring.

***DPYD* Allele**	**Wild-Type**	DPYD*2A	DPYD*13	DPYD*HapB3	**c.2846A>T**
**Wild-type**	GAS = 2	GAS = 1	GAS = 1	GAS = 1.5	GAS = 1.5
DPYD*2A		GAS = 0	GAS = 0	GAS = 0.5	GAS = 0.5
DPYD*13			GAS = 0	GAS = 0.5	GAS = 0.5
DPYD*HapB3				GAS = 1	GAS = 1
**c.2846A>T**					GAS = 1

DPYD*2A defined by rs3918290; c.2846A>T defined by rs67376798; DPYD*13 defined by rs55886062; DPYD*HapB3 defined by rs56038477; GAS: Gene (*DPYD*) Activity Score.

**Table 3:** CPIC and DPWG PGx guidelines recommendations for *DPYD*/fluoropyrimidine drug–gene interactions.

PGx Guideline	*DPYD* Variant	Phenotype	Recommendation
CPIC	*2A c.2846A>T *13 *HapB3 Others ^	GAS = 2 (NM)	Use the standard dose
		GAS = 1.5 (IM)	50% dose reduction followed by dose titration, based on clinical judgment and ideally on therapeutic drug monitoring
		GAS = 1 (IM)	50% dose reduction followed by dose titration, based on clinical judgment and ideally on therapeutic drug monitoring. In patients homozygous for c.2846A>T, a dose reduction of more than 50% may be required
		GAS = 0.5 (PM)	Alternative drug. If no other therapeutic option is available, strongly reduce the dose with early therapeutic drug monitoring
		GAS = 0 (PM)	Alternative drug
DPWG	*2A c.2846A>T *13 *HapB3	GAS = 2	Use the standard dose
		GAS = 1.5	Start with 50% of the standard dose or avoid fluorouracil and capecitabine. Adjust the subsequent doses guided by toxicity and effectiveness
		GAS = 1	Start with 50% of the standard dose or avoid fluorouracil and capecitabine. Adjust the subsequent doses guided by toxicity and effectiveness
		GAS = 0	Avoid fluorouracil and capecitabine. If not possible, determine DPD activity and adjust the dose

PGx: pharmacogenetic; CPIC: Clinical Pharmacogenetics Implementation Consortium; DPWG: Dutch Pharmacogenomics Working Group; GAS: Gene Activity Score; NM: normal metabolizer; IM: intermediate metabolizer; PM: poor metabolizer. ^ See genetic variants in supplementary Table S1 of the CPIC Guideline for fluoropyrimidines and DPYD [18].

**Table 4:** Baseline characteristics of fluoropyrimidine-treated patients.

Parameter	N = 167 n (%) or Mean ± sd
Women	62 (37.13)
Age	64.26 ± 10.89
BMI	26.69 ± 4.92
BS	1.80 ± 0.19
Ethnicity (European)	167 (100)
Tumor Location	
Colorectal	126 (75.45)
Gastric	13 (7.78)
Pancreas	10 (5.99)
Breast	12 (7.19)
Others	6 (3.59)
Tumor stage	
I	3 (1.80)
II	29 (17.36)
III	64 (38.32)
IV	71 (42.51)
Chemotherapy treatment	
Capecitabine (monotherapy)	72 (43.11)
XELOX	39 (23.35)
FOLFOX	40 (23.95)
FLOT	6 (3.59)
FOLFIRINOX	10 (5.99)
Initial doses (1000 mg/m^2^)%	
100	143 (85.63)
<100 and >50	18 (10.78)
50	6 (3.59)
Associated antibody	16 (9.58)
Toxicity endpoint	48 (28.74)
Efficacy endpoint	127 (71.86)
*DPYD* genotype	
DPYD*1/*1	161 (96.40)
DPYD*1/*HapB3	3 (1.80)
DPYD*1/rs67376798	2 (1.20)
DPYD*1/*13	1 (0.60)
DPD phenotype	
NM (GAS: 2)	161 (96.40)
IM (GAS: 1.5 or GAS:1)	6 (3.60)
PM (GAS: 0)	0 (0.00)

BMI: body mass index; BS: body surface; XELOX: capecitabine and oxaliplatin; FOLFOX: 5-fluorouracil, leucovorin, and oxaliplatin; FLOT: 5-fluorouracil, leucovorin, oxaliplatin, and docetaxel; FOLFIRINOX: 5-fluorouracil, leucovorin, irinotecan, and oxaliplatin; NM: normal metabolizer; IM: intermediate metabolizer; PM: poor metabolizer; GAS: Gene Activity Score.

**Table 5:** Association study of *DPYD* variants used for fluoropyrimidine dose tailoring with the response.

		**ADE Severity ≥ 3**		**OR (95% CI)**	***p*-Value**
		**Yes n (%)**	**NO n (%)**		
*DPYD* GAS < 2	YES n (%)	2 (4.3)	4 (3.3)	1.29 (0.11–9.34)	0.674
	NO n (%)	45 (95.7)	116 (96.7)		
		**Efficacy**		**OR (95% CI)**	***p*-Value**
		**Yes n (%)**	**NO n (%)**		
*DPYD* GAS < 2	YES n (%)	3 (2.3)	3 (7.9)	0.28 (0.04–2.19)	0.132
	NO n (%)	126 (97.7)	35 (92.1)		

ADE: adverse drug event; OR = odds ratio; CI: confidence interval; GAS = Gene Activity Score.

**Table 6:** Association study with the efficacy/toxicity endpoints of *DPYD* variants not used for FP dose tailoring.

		**ADE Severity ≥ 3**		**OR (95% CI)**	***p*-Value**
		**Yes n (%)**	**NO n (%)**		
*DPYD* rs1801265	A/G–G/G	15 (31.9)	38 (31.9)	1.00 (0.48–2.06)	1
	A/A	32 (68.1)	81 (68.1)		
*DPYD* rs17376848	A/G	0 (0)	6 (5)	0.00 (0–NA)	0.043
	A/A	47 (100)	113 (95)		
*DPYD* rs1801159	C/T–T/T	18 (38.3)	53 (44.5)	0.77 (0.39–1.54)	0.46
	C/C	29 (61.7)	66 (55.5)		
*DPYD* rs1801160	C/T	6 (12.8)	15 (12.6)	1.01 (0.37–2.80)	0.98
	C/C	41 (87.2)	104 (87.4)		
*DPYD* rs1801158	C/T	6 (12.8)	3 (2.5)	5.66 (1.35–23.67)	0.014
	C/C	41 (87.2)	116 (97.5)		
*DPYD* rs2297595	C/T	11 (23.4)	24 (20.2)	1.21 (0.54–2.72)	0.65
	C/C	36 (76.6)	95 (79.8)		
		**Efficacy**		**OR (95% CI)**	***p*-Value**
		**Yes n (%)**	**NO n (%)**		
*DPYD* rs1801265	A/G–G/G	41 (31.8)	12 (32.4)	0.97 (0.44–2.12)	0.94
	A/A	88 (68.2)	25 (67.6)		
*DPYD* rs17376848	A/G	6 (4.7)	0 (0)	NA (0.00-NA)	0.079
	A/A	123 (95.3)	37 (100)		
*DPYD* rs1801159	C/T–T/T	56 (43.4)	15 (40.5)	1.13 (0.54–2.37)	0.76
	C/C	73 (56.6)	22 (59.5)		
*DPYD* rs1801160	C/T	16 (12.4)	5 (13.5)	0.91 (0.31–2.66)	0.86
	C/C	113 (87.6)	32 (86.5)		
*DPYD* rs1801158	C/T	6 (4.7)	3 (8.1)	0.55 (0.13–2.33)	0.43
	C/C	123 (95.3)	34 (91.9)		
*DPYD* rs2297595	C/T	28 (21.7)	7 (18.9)	1.19 (0.47–2.99)	0.71
	C/C	101 (78.3)	30 (81.1)		

ADE: adverse drug event; OR = odds ratio; CI: confidence interval.

**Table 7:** Association study with the toxicity and efficacy endpoint of study variables included in the multivariate model after adjustment.

**Toxicity Endpoint**		
**Variable**	**OR (95% CI)**	***p*-Value**
*DPYD* rs1801158 (CT or TT)	5.73 (1.41–28.77)	0.019
Irinotecan	2.32 (0.92–5.81)	0.071
Age	NA	0.090
*DPYD* rs56038477 (CT or TT)	6.99 (0.64–155.45)	0.120
Monoclonal antibody	0.00 (NA–Inf)	0.990
*DPYD* rs17376848 (AG or GG)	0.00 (NA–Inf)	0.992
**Efficacy Endpoint**		
**Variable**	**OR (95% CI)**	***p*-Value**
Irinotecan	0.15 (0.06–0.35)	<0.001
*DPYD* rs56038477 (CT or TT)	0.096 (0.01–1.05)	0.062
Radiotherapy	2.31 (0.79–8.48)	0.16

ADE: adverse drug event; OR= odds ratio; CI: confidence interval; NA: not applicable.

**Table 8:** *DPYD* characterization in our population.

*Allele	*DPYD* Variant	Major Nucleotide Variation	Genotype N = 190 ^			H-W	MAF	MAF Ibs	Comparison with 1000 Genomes
			*Wt*	*Het*	*Hom*				
-	rs17376848	c.1896T>C	181 (95.77)	8 (4.23)	0 (0)	1	0.021	98/2	1
*4	rs1801158	c.1601G>A	177 (93.65)	12 (6.35)	0 (0)	1	0.032	94/6	0.149
*5	rs1801159	c.1627A>G	109 (57.67)	69 (36.51)	11 (5.82)	1	0.241	79/21	0.475
*6	rs1801160	c.2194G>A	168 (88.89)	21 (11.11)	0 (0)	1	0.056	95/5	0.643
*9A	rs1801265	c.85T>C	128 (67.73)	58 (30.69)	3 (1.59)	0.3	0.169	79/21	0.170
-	rs2297595	c.496A>G	152 (80.42)	37 (10.58)	0 (0)	0.2	0.098	88/12	0.470
*2A	rs3918290	c.1905+1G>A	190 (100)	0 (0)	0 (0)	-	0	100/0	1
*13	rs55886062	c.1679T>G	189 (99.47)	1 (0.53)	0 (0)	1	0.003	100/0	1
*HapB3	rs56038477	c.1236G>A	185 (97.37)	5 (2.63)	0 (0)	1	0.013	98/2	0.729
-	rs67376798	c.2846A>T	188 (98.95)	2 (1.05)	0 (0)	1	0.011	100/0	0.538

Wt: wild-type; Het: heterozygous; Hom: recessive homozygous; H-W: Hardy–Weinberg equilibrium analysis; MAF: minor allele frequency; Ibs: Iberian Peninsula; * MAFs obtained from the 1000 Genomes Project [15]. ^ N = 189 for *DPYD* variants not used for fluoropyrimidine dose tailoring.

**Table 9:** Linkage disequilibrium analysis.

Linkage Disequilibrium									
n = 190 ^									
	rs1801265 (*9A)	**rs17376848**	rs1801159 (*5)	rs1801160 (*6)	rs1801158 (*4)	**rs2297595**	rs55886062 (*13)	**rs67376798**	rs56038477 (*HapB3)
rs1801265 (*9A)	-	0.9463	0.6052	0.0369	0.1844	0	0.0272	0.5667	1 × 10^−4^
		2 × 10^−4^	−0.0045	0.0103	−0.0044	0.0684	0.0024	−9 × 10^−4^	0.0074
		0.012	0.1142	0.1943	0.9901	0.7748	0.9751	0.9191	0.9903
		0.0037	−0.0284	0.1145	−0.0728	0.6035	0.1212	−0.0314	0.2139
**rs17376848**	-	-	0.8753	0.5391	0.7111	0.6804	0.9834	0.9584	0.897
			5 × 10^−4^	−0.0011	−4 × 10^−4^	−9 × 10^−4^	0	0	−1 × 10^−4^
			0.0358	0.9562	0.8977	0.4854	0.0028	0.2721	0.5501
			0.0086	−0.0337	−0.0203	−0.0226	0.0011	−0.0029	−0.0071
rs1801159 (*5)	-	-	-	0.9568	0.0887	0.0141	0.0848	0.9315	0.2636
				−3 × 10^−4^	−0.0065	−0.0177	0.0022	2 × 10^−4^	0.0025
				0.0203	0.9933	0.6966	0.9725	0.0342	0.3631
				−0.003	−0.0934	−0.1347	0.0946	0.0047	0.0613
rs1801160 (*6)	-	-	-	-	0.4411	0.6994	0.8619	0.0191	0.1098
					0.0017	0.0016	−1 × 10^−4^	0.0024	0.002
					0.9746	0.2376	0.6704	0.4308	0.2396
					−0.0423	−0.0212	−0.0095	0.1287	0.0878
rs1801158 (*4)	-	-	-	-	-	0.3038	0.9693	0.9033	0.8394
						−0.0028	0	−1 × 10^−4^	−2 × 10^−4^
						0.9848	0.231	0.5147	0.7001
						−0.0564	−0.0021	−0.0067	−0.0111
**rs2297595**	-	-	-	-	-	-	0.7833	0.6709	0.4257
							−3 × 10^−4^	−6 × 10^−4^	0.0013
							0.8023	0.8752	0.1577
							−0.0151	−0.0233	0.0437
rs55886062 (*13)	-	-	-	-	-	-	-	0.8471	0.8998
								0	0
								0.0149	0.012
								0.0106	0.0069
**rs67376798**	-	-	-	-	-	-	-	-	0.95
									0
									0.0042
									0.0034
rs56038477 (*HapB3)	-	-	-	-	-	-	-	-	*p*-value
									D
									D’
									r

^ For rs1801265 (*9A), rs17376848, rs1801159 (*5), rs1801160 (*6), rs1801158 (*4), rs2297595. n = 189 patients were genotyped, because we did not have enough DNA for n = 1 patient. Green data: linkages with *p*-value < 0.05. For each pair of genetic variants, we report, from top to bottom, the *p*-value, D, D’, and r values.

## Data Availability

The data presented in this study are available on request from the corresponding author. The data are not publicly available due to containing clinical and personal information.
